# Antimicrobial Materials with Lime Oil and a Poly(3-hydroxyalkanoate) Produced via Valorisation of Sugar Cane Molasses

**DOI:** 10.3390/jfb11020024

**Published:** 2020-04-10

**Authors:** Pooja Basnett, Elena Marcello, Barbara Lukasiewicz, Rinat Nigmatullin, Alexandra Paxinou, Muhammad Haseeb Ahmad, Bhavana Gurumayum, Ipsita Roy

**Affiliations:** 1 Faculty of Science and Technology, University of Westminster, London W1W 6UW, UK; P.Basnett@westminster.ac.uk (P.B.); w1614733@my.westminster.ac.uk (E.M.); barbara.lukasiewicz@gmail.com (B.L.); rn17541@bristol.ac.uk (R.N.); w1621777@my.westminster.ac.uk (A.P.); Mhaseebahmadd@gmail.com (M.H.A.); gurumayumbhavana@gmail.com (B.G.); 2 Bristol Composites Institute (ACCIS), University of Bristol, Bristol BS8 1TR, UK; 3 Department of Material Science and Engineering, Faculty of Engineering, University of Sheffield, Sheffield S1 3JD, UK

**Keywords:** polyhydroxyalkanotes, sugarcane molasses, antibacterial materials, essential oils

## Abstract

A medium chain-length polyhydroxyalkanoate (PHA) was produced by *Pseudomonas mendocina* CH50 using a cheap carbon substrate, sugarcane molasses. A PHA yield of 14.2% dry cell weight was achieved. Chemical analysis confirmed that the polymer produced was a medium chain-length PHA, a copolymer of 3-hydroxyoctanoate and 3-hydroxydecanoate, P(3HO-*co*-3HD). Lime oil, an essential oil with known antimicrobial activity, was used as an additive to P(3HO-*co*-3HD) to confer antibacterial properties to this biodegradable polymer. The incorporation of lime oil induced a slight decrease in crystallinity of P(3HO-co-3HD) films. The antibacterial properties of lime oil were investigated using ISO 20776 against *Staphylococcus aureus* 6538P and *Escherichia coli* 8739, showing a higher activity against the Gram-positive bacteria. The higher activity of the oil against *S. aureus* 6538P defined the higher efficiency of loaded polymer films against this strain. The effect of storage on the antimicrobial properties of the loaded films was investigated. After one-year storage, the content of lime oil in the films decreased, causing a reduction of the antimicrobial activity of the materials produced. However, the films still possessed antibacterial activity against *S. aureus* 6538P.

## 1. Introduction

Polyhydroxyalkanoates (PHAs) are a family of natural polymers produced by bacterial fermentation using renewable carbon sources under nutrient-limiting conditions. PHAs can be broadly classified into short chain length (SCL: C_3_-C_5_) and medium-chain length (MCL: C_6_-C_14_) based on the number of carbon atoms within their monomer units [1]. SCL PHAs have a high melting temperature and are brittle, whereas MCL PHAs are elastomeric and have a low-melting and glass-transition temperature. PHAs can match the functional properties of common petroleum-based plastics. For example, the mechanical properties of poly(3-hydroxybutyrate) (P(3HB)) are similar to those of polystyrene, while its copolymers with 3-hydroxyvalerate (P(3HB-*co*-3HV)) are characterised by an improved ductility resulting in a combination of mechanical properties close to polypropylene. One the other hand, MCL-PHAs can emulate the properties of soft thermoplastic elastomers such as thermoplastic polyurethanes, olefinic thermoplastic elastomers. The functional properties of PHA-based materials can be further modified and tuned via blending with other polymers [2]. Thanks to the presence of hydrolysable ester linkage connecting the monomer units of PHAs, PHAs are biodegradable in nature unlike most of conventional plastics. Their degradation rate is affected by the chemical composition of the polymer, and by environmental factors [3]. Based on life-cycle assessment (LCA) analysis, PHAs have been recognised as one of the safest bioplastics for the environment [4]. PHAs are also known to be biocompatible and have been widely investigated for various biomedical applications [5].

Although PHAs represent a family of sustainable polymers with attractive properties, they are yet to replace petroleum based conventional plastics used in most industrial applications. One of the major factors limiting the commercialisation of PHAs is its high production cost. Several cheap carbon sources, waste materials, genetically modified bacterial strains, fermentation conditions and recovery methods have been employed either on their own or in combination, to allow economical production of PHAs [3]. Since the cost of carbon accounts for 31% of the overall PHA production cost, the use of inexpensive substrates is one of the effective strategies to produce PHAs in an economical manner [6,7]. Simple sugars, triglycerides (vegetable oils) are the most widely used carbon sources for the microbial production of PHAs [8]. In addition to their high cost, the utilization of such raw materials can be in competition with the use of these resources as food. Therefore, the valorisation of cheap agri-food by-products can address both issues; the cost of carbon source and the competition with food. Although potentially biomass rich in polysaccharides can be used for generating simple sugars, this requires additional chemical and enzymatic treatment. Molasses and whey, on the other hand, contain up to 60 and 4.5 wt.% of simple sugars, respectively. Currently, a significant part of molasses is used in bioethanol production. The production of PHAs with the use of molasses as the carbon feedstock is a feasible alternative for the valorisation of molasses. Many studies demonstrated that molasses is a good substrate for microbial production of poly(3-hydroxybutyrate) [9].

PHAs have diverse physical properties which makes them suitable for various applications such as packaging, medical devices, bio-implants and tissue engineering [10]. For all these applications, microbial contamination is a major issue. Bacterial adhesion and colonisation on the polymer surface form a reservoir for pathogens which leads to various nosocomial infections, skin related issues, degradation of food and cosmetic products, and food contamination [11]. For several decades, antibiotics have been used to treat and prevent such infections. However, with the rise in the number of multidrug-resistant pathogens, antibiotic resistance has now become a global health risk. Annual costs of treating antibiotic resistant infections is estimated to be between $21,000 and $34,000 million in the United States alone and around €1500 million in Europe [12]. In such a scenario, there is a high demand for natural antibacterial agents to prevent or mitigate bacterial contamination.

Traditionally, essential oils, plant extracts and other natural products were used to treat infectious diseases. According to the World Health Organization (WHO), the majority of the world population is dependent on treatment using natural products as a primary care. Various essential oils have shown to possess antibacterial, antifungal and antioxidant properties [13,14,15]. These oils are now being screened to find alternative remedies to fight bacterial infections in a wide range of applications. Generally, a higher antimicrobial activity has been observed for oxygenated constituents of essential oils [16]. However, as antimicrobial additives to polymer materials, non-polar terpene hydrocarbons can bring other benefits such as improved compatibility with hydrophobic polymer matrices, enhanced water vapour barrier properties and plasticising effect [17]. Lime oil is an example of essential oils rich of terpene hydrocarbons such as limonene (ca. 40%), β-pinene (ca. 25%), and γ-terpinene (ca. 10%) [18]. The antimicrobial activity of lime oil is well documented [18,19,20].

The aim of this study was the development of natural antimicrobial polymer films via the incorporation of an essential oil into biodegradable PHA produced from agri-food by-products. We report a feasible way of valorisation of sugarcane molasses for the production of PHA by *Pseudomonas mendocina* CH50, a bacterial strain which has been previously confirmed as an efficient producer of MCL PHAs [5,20,21]. It was demonstrated that in the presence of sugarcane molasses *P. mendocina* CH50 was able to accumulate an MCL PHA, which was identified as a copolymer of 3-hydroxyoctanoate and 3-hydroxydecanoate, or P(3HO-*co*-3HD). Lime oil was incorporated into P(3HO-*co*-3HD) films to produce a natural antimicrobial material. The oil content in the films was monitored during long storage, and loss of oil was observed during the storage. Both fresh and aged films exhibited antimicrobial activity against Gram-positive *S. aureus*. The films were less efficient against Gram-negative *E. coli* reflecting lower activity of lime oil against this bacterial strain.

## 2. Material and Methods

### 2.1. Bacterial Strains and Culture Conditions

*Pseudomonas mendocina* CH50 was obtained from the University of Westminster culture collection. The strain was cultured at 30 °C for 16 h in a shaking incubator (200 rpm). After incubation, the strain was stored as a glycerol stock at −80 °C. For the antibacterial characterization, *Staphylococcus aureus* 6538P and *Escherichia coli* 8739 were bought from the American Type Culture Collection (ATCC). They were cultured in sterile nutrient broth at 37 °C and 200 rpm for 16 h in a shaking incubator and stored as a glycerol stock at −80 °C.

### 2.2. Chemicals

All the chemicals for the production and characterization of PHAs were purchased from Sigma Aldrich Ltd., England and VWR international, England. Lime oil used as an antibacterial agent was purchased from Holland and Barrett, London, UK.

### 2.3. Production of Polyhydroxyalkanoates by Pseudomonas mendocina *CH50* Using Sugarcane Molasses as the Sole Carbon Source

Production of PHAs by *Pseudomonas mendocina* CH50 using 20 g/L of sugarcane molasses was carried out in 15 L bioreactors, with 10 L working volume (Applikon Biotechnology, Tewkesbury, UK). Batch fermentation was carried out in two stages. The seed culture was prepared using a single colony of *P. mendocina* CH50 to inoculate sterile nutrient broth. This was incubated for 16 h at 30 °C and at 200 rpm. Inoculum of 10 vol.% of the final volume was used to inoculate the second stage seed culture (mineral salt medium-MSM) which was incubated at 30 °C, 200 rpm for 24 h. Second stage seed culture was used to inoculate the final PHA production media [21,22]. The culture was grown for 48 h, at 30 °C, 200 rpm and air flow rate of 1 vvm. Temporal profile of the production of PHAs by *P. mendocina* CH50 using 20 g/L sugarcane molasses was obtained by monitoring parameters such as optical density, biomass, nitrogen and glucose concentrations, pH and dissolved oxygen tension (DOT) at regular intervals during the fermentation.

### 2.4. Analytical Studies

Bacterial growth was measured by monitoring the optical density (OD) at 450 nm. Biomass yield was determined by weighing the dried pellet of the cells obtained after centrifugation of 1 mL at 12,000 rpm for 10 min (Heraeus Pico 17 Centrifuge, Thermofisher Scientific, MA, USA). pH of the supernatant was measured using Seven Compact pH meter (Mettler Toledo Ltd., Leicester, UK). Nitrogen in the form of ammonium ions was estimated by the phenol-hypochlorite method [21,22].

Polymer was extracted from the dried biomass using the soxhlet extraction method. To remove organic soluble impurities, dried biomass was refluxed in methanol for 24 h followed by refluxing in chloroform for 48 h to extract the polymer. Polymer solution in chloroform was concentrated using a rotary evaporator. Polymer was precipitated using ice-cold methanol under continuous stirring and dried at room temperature. Polymer yield was calculated as a percentage of dry cell weight (DCW), using the formula:

(1) $$\%\mathrm{DCW}=\frac{\mathrm{Polymer}\;\mathrm{mass}}{\mathrm{Mass}\;\mathrm{of}\;\mathrm{biomass}}\times 100$$

### 2.5. Preparation of Film Samples

Solvent cast films were prepared by pouring 5 wt.% polymer solution in chloroform in 60 mm diameter glass Petri dishes. The polymer was dried in the fume hood at the room temperature until the solvent evaporated completely. For oil-incorporated films, the oils were added to 5 wt.% polymer solution in chloroform. The loaded films were stored at room temperature in sealed Petri dishes for one year at room temperature and identified as aged samples. Quantification of the oil content in the films after complete solvent evaporation was carried out by thermogravimetric analysis (TGA) using the STA 449 F3 Jupiter^®^ instrument (Netzsch, Germany); 10–15 mg film samples were heated at 10 degrees per minute from 30 to 150 °C under nitrogen flow and kept at 150 °C for 2 h. The cumulative mass loss in dynamic and isothermal steps was defined as oil amount left in the films after solvent evaporation.

### 2.6. Polymer Characterization

The monomeric composition of polymer was determined using gas chromatography–mass spectrometry (GC–MS) analysis and ^13^C, ^1^H nuclear magnetic resonance (NMR) spectroscopy. For GC–MS analysis (Chrompack CP-3800 gas chromatograph and Saturn 200 MS/MS block), 20 mg of the polymer sample was subjected to methanolysis as described in Basnett et al. [5]. For NMR analysis, the polymer was dissolved in deuterated chloroform (20 mg/mL).

Thermal analysis of polymers was conducted using a differential scanning calorimeter (DSC) 214 Polyma (Netzsch, Germany) equipped with Intracooler IC70 cooling system. 5 mg of polymer was heated from −50 to 100 °C at a heating rate of 20 °C per min. DSC thermograms were analysed using Proteus 7.0 software.

### 2.7. Antibacterial Characterization

#### 2.7.1. MIC (Minimum Inhibition Concentration) and MBC (Minimum Bactericidal Concentration)

The minimum inhibition concentration (MIC) was estimated according to the ISO 20776 against *Staphylococcus aureus* 6538P and *Escherichia coli* 8739. Stock solution of the lime oil was prepared in Mueller Hinton Broth and 1% dimethyl sulfoxide (added as a co-solvent which enables mixing the oils with aqueous medium). A range of concentrations from 100 to 5 µL/mL essential oils was tested in a 96-well plate. Each well was inoculated to a final volume of 100 µL using a microbial suspension adjusted to achieve a final concentration of 5 × 10^5^ CFU/mL and incubated at 37 °C for 24 h at 100 rpm. Studies were performed in triplicate. The MIC was determined by measuring the absorbance at 600 nm. Following the MIC test, the entire volume of the MIC well and the concentrations above were removed and 10-fold dilution was performed in PBS and plated onto agar plates. The plates were incubated at 37 °C for 24 h. The minimum bactericidal concentration (MBC) was determined as the lowest concentration of the compound that induced 99.9% killing of the bacteria.

#### 2.7.2. Halo Test

The films loaded with essential oils were tested for antibacterial activity using the halo test against *Staphylococcus aureus* 6538P and *Escherichia coli* 8739. The test was conducted according to the European Committee on Antimicrobial Susceptibility Testing (EUCAST) Disc Diffusion Test Methodology. A single colony was used to prepare a suspension of 0.5 McFarland turbidity standard (approx. 10^8^ colony-forming units (CFU)/mL). A sterile cotton swab was immersed into the suspension and was spread evenly on the agar plates. The agar plates as well as the antimicrobial discs were kept at room temperature prior to inoculation. Polymer films of 0.7 cm diameter containing active agents, sterilized under ultraviolet (UV) light for 30 min (15 min each side) were placed on the inoculated agar surface. The plates were incubated at 37 °C for 18 h. The inhibition zones formed were measured and noted. For the antibacterial test, both freshly prepared samples and aged samples were investigated. Each material was tested in triplicates. Films without active agents were used as negative controls, antibiotic discs containing oxacillin (1 μg/disc) and streptomycin (300 μg/disc) were used as positive controls against *Staphylococcus aureus* 6538P and *Escherichia coli* 8739, respectively.

## 3. Results and Discussion

### 3.1. Temporal Profile of Polyhydroxyalkanoate (PHA) Production by Pseudomonas mendocina *CH50* Using Sugarcane Molasses as the Sole Carbon Source

**Figure 1:**
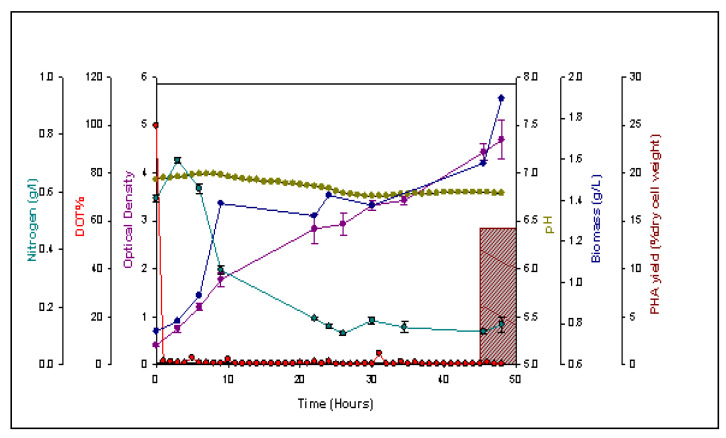
Temporal profile of P(3HO-*co*-3HD) production by *P. mendocina* CH50 using sugarcane molasses as the sole carbon source. The profiling illustrates the polyhydroxyalkanoate (PHA) yield (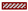), optical density (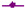), pH (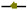), DOT% (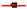), biomass (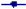) and the nitrogen concentration in the form of the ammonium ions throughout 48 h of fermentation.

Figure 1 represents the fermentation profile of the PHA production by *P. mendocina* CH50 using sugarcane molasses as the carbon source in a 15 L bioreactor. Fermentation was performed as described in Section 2.3., and OD, pH, % DOT, biomass, nitrogen concentration and polymer yield were monitored.

The optical density increased gradually until the first 3 h (lag phase) of the fermentation and continued to increase steadily until the end of process, reaching a value of 4.7. Biomass concentration increased until 48 h reaching a value of 1.9 g/L. Prior to the fermentation, pH of the production media was set to 7.0. The pH of the culture did not change significantly during the fermentation process whereas the DOT% reduced from 100% to 0% within few hours of the fermentation and remained very low until the end of the process, maintaining an oxygen-limiting environment. Nutrient limitation plays an important role in the production of PHAs. Furthermore, it is known that nutrient limitation coupled with an excess of carbon provides an ideal environment to produce PHAs [23]. In this study, nitrogen was the limiting factor. As illustrated in Figure 1, the nitrogen concentration decreased from 0.58 g/L to 0.14 g/L at the end of 48-h fermentation. The PHA yield (% dcw) at 48 h was found to be was 14.22%. As mentioned above, optical density and biomass concentration increased steadily during the fermentation indicating cell growth in the presence of nitrogen in the media. The absence of nitrogen limitation could have influenced the overall accumulation of PHAs. Sugarcane molasses have been previously used as a carbon substrate to produce SCL-PHA by *Bacillus cereus* SPV with a PHA yield of 51.37% dcw [24]. In another study, Naheed and Jamil obtained a maximum PHA yield of 80.5% dcw by *Enterobacter* sp. SEL2, a bacterium isolated from soil of contaminated sites, with the use 3% of sugarcane molasses after fermentation for 24 h at pH 5.0 [25]. Chaijamrus and Udpuay reported a 43% yield of P(3HB) by *Bacillus megaterium* ATCC 6748 after 45 h of fermentation when 4% sugarcane molasses was used as the carbon source [26]. With regards to MCL-PHA production using sugarcane molasses, a limited investigation has been conducted. This is due to the fact that the majority of the *Pseudomonas* species (i.e., the main producer of MCL-PHAs) cannot utilize sucrose, which is one of the main components of sugarcane molasses along with glucose and fructose [27]. Therefore, the substrate is usually modified in order to obtain equimolar concentrations of glucose and fructose by either ionic exchange or acid hydrolysis [27]. To date, only two species of the *Pseudomonas* family have been shown to metabolize sucrose directly, *P. fluorescens* A2a5 and *P. corrugata* 388.

*P. fluorescens* A2a5 produced P(3HB) with a yield of 70% dcw when cultured using sugarcane liquor [28]. In contrast, Solaiman *et al*. showed that *P. corrugata* 388 was able to consume sucrose (present in soy molasses used as carbon source) producing an MCL-PHA with a yield of 17% dcw [27]. *P. mendocina* CH50, the strain used in this study is not able to utilize the sucrose present in the media. The PHA produced by *P. mendocina* CH50 using sugarcane molasses as the carbon source was further characterised.

### 3.2. Polymer Characterisation

#### 3.2.1. Polymer Composition Analysis

**Figure 2:**
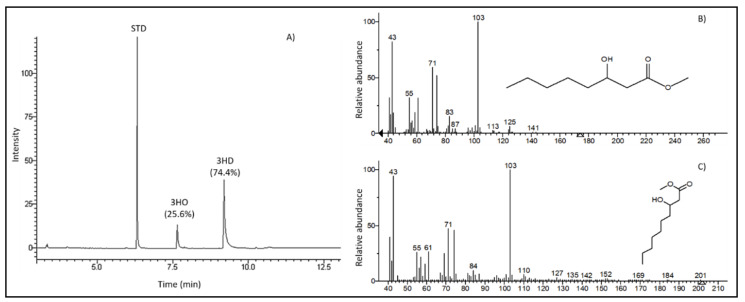
Gas chromatography–mass spectrometry (GC–MS) data of the PHA copolymer produced with sugarcane molasses as the carbon source: (**A**) Gas chromatogram, (**B**) Mass spectrum of a peak with R_t_ = 7.7 min identified using the National Institute of Standards and Technology (NIST) library as methyl ester of 3-hydroxyoctanoic acid, and (**C**) mass spectrum of a peak with R_t_ = 9.2 min identified using NIST library as methyl ester of 3-hydroxydecanoic acid. Methyl benzoate was used as an internal standard (STD).

As can be seen from Figure 2, GC–MS chromatograms of the methanolysed polymer demonstrated two peaks identified using the National Institute of Standards and Technology (NIST) library. The retention peak at 7.7 and 9.2 min were identified as the methyl ester of 3-hydroxyoctanoic acid and methyl ester of 3-hydroxydecanoic acid respectively. Peak at 6.64 min corresponded to the methyl benzoate, used as an internal standard. Thus, the polymer produced by *P. mendocina* CH50 using sugarcane molasses as the sole carbon source was identified as the copolymer of 3-hydroxyoctanoate and 3-hydroxydecanoate, Poly(3-hydroxyoctanoate-*co*-3-hydroxydecanoate) or P(3HO-*co*-3HD). The mole percentage of 3-HO and 3-HD was found to be 25.6% and 74.4% respectively. P(3HO-co-3HD) copolymers with 3-HD-dominant composition are usually produced by *Pseudomonas* species when grown on structurally unrelated simple sugars [29].

**Figure 3:**
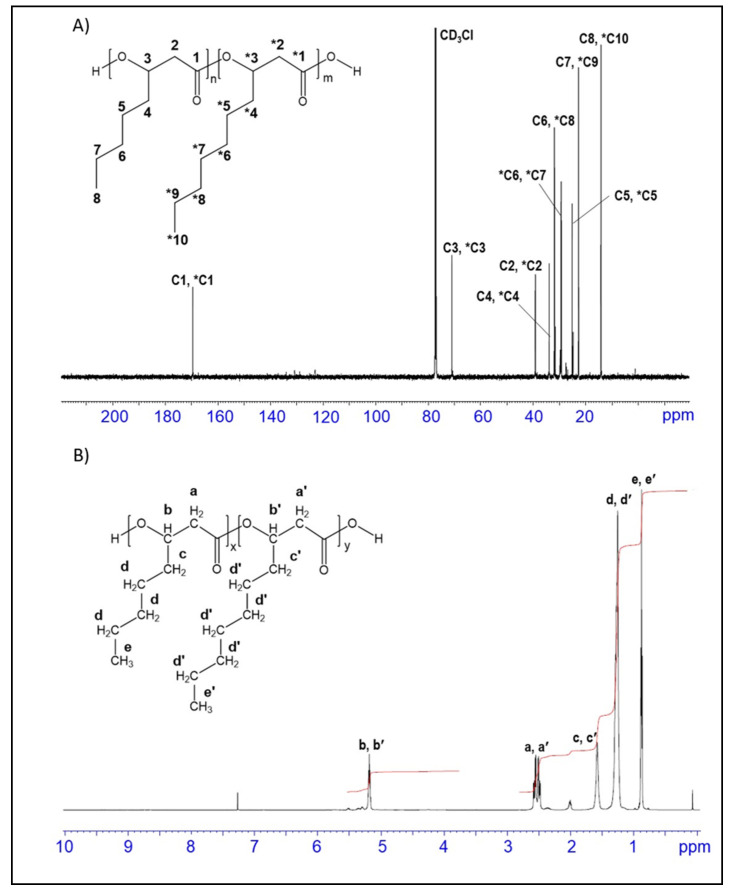
^13^C nuclear magnetic resonance (NMR) (**A**), and ^1^H NMR (**B**) spectra of PHA copolymer produced with sugarcane molasses as carbon source. The structure of P(3HO-*co*-3HD) is shown as an insert within the spectra.

In order to confirm the structure of synthesised PHA, NMR analysis has been performed. ^1^H and ^13^C NMR spectra are presented in Figure 3. All peaks expected for P(3HO-*co*-3HD) where observed in ^1^H and ^13^C NMR spectroscopy. The chemical shift at 169, 70.9, 39.1 ppm corresponds to C_1_ (C=O), C_3_ (–CH), C_2_ (–CH_2_) respectively, forming the backbone of the copolymer. The resonance in the range between 23–35 ppm related to olefinic methylene groups: C_4_,*C_4_ (33.86, 33.92 ppm), C_5_, *C_5_ (24.84, 25.20 ppm), C_6_,*C_6_ (31.64, 29.48 ppm), C_7_,*C_7_ (22.63, 29.32 ppm), *C_8_ (31.91 ppm), *C_9_ (22.76 ppm) (carbon atoms of 3-hydroxydecanoate market with asterisk). The terminal methyl groups (–CH_3_) of C_8_, *C_10_ were observed at 14.12 and 14.22 ppm, respectively. All observed peaks in the ^13^C spectrum (Figure 3A) are in agreement with spectral characteristic reported for 3-hydroxyoctanoate and 3-hydroxydecanoate [30].

The ^1^H NMR spectrum (Figure 3B) shows 5 groups of overlapping peaks corresponding to 5 different proton environments characteristic for medium chain 3-hydroxyalakanoates. Three different environments of the olefinic proton manifested at chemical shifts 0.8, 1.2, and 1.5 ppm. The chemical shift at 0.8 corresponds to the methyl groups of both monomers. The protons of the methylene group at carbon atoms 5, 6, 7 of 3-hydroxyoctanoate and carbon atoms 5, 6, 7, 8, 9 of 3-hydroxydecanoate were observed at 1.2 ppm, while proton resonance of the methylene group at carbon 4 for both monomers shifted further downfield to 1.5 ppm. Two types of backbone protons for both monomers generated signals at chemical shifts of 2.52 (methylene group of carbon 2) and 5.2 (methine group of carbon 3) ppm.

The monomeric composition of PHAs is strictly influenced by the carbon source utilized. In presence of structurally related carbon sources (e.g., fatty acids), the final composition of the polymer reflects that of the substrate. When unrelated substrates (e.g., carbohydrates) are utilized, the monomeric unit of the PHAs are independent of the structure of the carbon source [1,2,3]. As mentioned before, sugarcane molasses is composed of up to 60% of sugars which are classified as unrelated substrates. *Pseudomonas* sp. can convert sugars into PHAs through the *de novo* fatty acid pathway. One of the enzymes involved in this pathway has a high affinity to the substrate containing 10 carbon atoms [31]. For this reason, *Pseudomonas* sp. have been shown to produce PHAs with a high content of the 3-HD monomer when cultured using glucose or glycerol [5,31]. This study is in agreement with previous studies, as the MCL-PHA produced showed a high content of 3-HD.

#### 3.2.2. Properties of Copolymer and Films Containing Lime Oil

**Figure 4:**
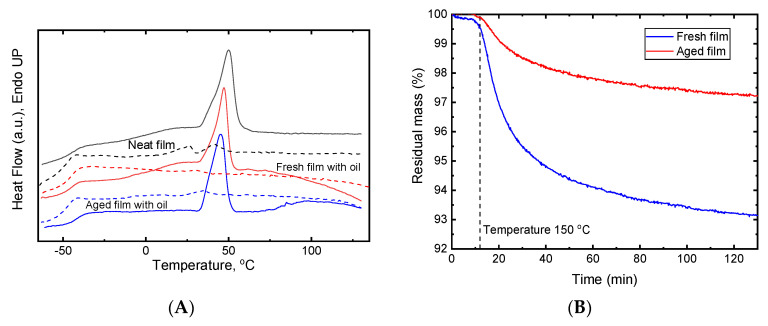
Thermal analysis of P(3HO-*co*-3HD) neat film and films with incorporated lime oil. (**A**) Differential scanning calorimetry (DSC) thermograms: neat film (black lines), fresh film with lime oil (red lines), and aged films with lime oil (blue lines). First heating—solid line, second heating—dashed line. Thermograms were shifted vertically for better visibility. (**B**) Thermogravimetric analysis (TGA) curves for determination of oil content after isothermal evaporation at 150 °C for 2 h.

The DSC of P(3HO-*co*-3HD) produced with sugarcane molasses as the carbon source revealed both low glass transition and melting temperature (Figure 4A, Table 1) which are characteristic properties of MCL-PHAs [5]. With glass-transition temperature ca. −45 °C, the amorphous phase of this semi-crystalline polymer is in a rubbery state at room temperature and polymer is expected to be a flexible and stretchable material. Also, such low glass transition would prevent transition of the polymer into a brittle material at sub-zero temperatures which can be required for a packaging material. The melting temperature is around 55 °C. This could be beneficial for melt processing at relatively low temperatures. Melting is not observed in a DSC thermogram of second heating demonstrating slow kinetics of the crystallisation process for this copolymer.

Incorporating lime oil into P(3HO-*co*-3HD) films decreased the melting temperature by approximately 4 °C. For an evaluation of the effect of lime oil on the crystallinity of the copolymer, an observed enthalpy of melting of P(3HO-*co*-3HD) in films containing lime oil needs to be normalised to the actual mass fraction of the copolymer. The real content of oil in the films does not correspond to oil/polymer composition in the casting solution due to oil loss as a result of evaporation along with solvent during the film formation as well as during the storage of the prepared film. Residual amounts of lime oil in P(3HO-*co*-3HD) films were determined from TGA experiments (Figure 4B) and found to be 6.9 wt.% for the new film and 2.8 wt.% for the aged one. The enthalpy of melting of the P(3HO-*co*-3HD) crystalline phase decreased from 18.2 J/g to 14.7 and 15.8 J/g for fresh and aged films, respectively. Thus, the addition of lime oil induced a decrease in crystallinity of the copolymer. This implies that lime oil components had a plasticising effect on the copolymer. Slight increase in enthalpy of polymer melting (and thereby crystallinity degree) for aged films compared with the fresh one might be explained by continuation of crystallisation during 1-year storage and by promotion of crystallisation with the loss of plasticising lime oil. Also, with storage, enthalpy of melting shifted to higher temperatures indicating rigidification of the amorphous phase.

### 3.3. Antibacterial Characterization

#### 3.3.1. MIC (Minimum Inhibition Concentration) and MBC (Minimum Bactericidal Concentration)

Lime oil was tested against *S. aureus* 6538P and *E. coli* 8739, chosen as an example of Gram-positive and Gram-negative bacteria, respectively. The values obtained are shown in Table 2. The MIC and MBC values against *S. aureus* 6538P were 60 and 100 µL/mL respectively. While against *E. coli* 8739, MIC value of 80 µL/mL was obtained, no MBC could be determined at the concentration investigated. The MBC is defined as the lowest concentration of compound inducing 99.9% killing of the bacteria, corresponding to 3 log reduction in CFU. Therefore, the results obtained for lime oil showed that the compound had a bacteriostatic rather than bactericidal effect against *E. coli* 8739 at the concentrations investigated. In literature, MIC and MBC values for lime oil and generally for all essential oils comprise a very large range. For lime oil MIC values were reported from 0.1 mg/mL [18] to few hundreds mg/mL [32]. Such wide variations can be caused by plant sources, time of harvesting, methods of oil extraction, etc. Lime oil used in this study was of relatively low antimicrobial activity.

The antibacterial mechanism of such essential oils is usually related to the activity of their main components, terpenes and terpenoids. Lime oil (*Citrus aurantifolia*) is composed mainly of limonene [18,20]. The mechanism of action of such organic compounds is linked to their ability to modify the integrity of the bacterial cell wall, increasing its permeability and causing membrane disruption [15,33]. Moreover, a higher effect against Gram-positive bacteria compared to negative ones is usually reported, due to protection of Gram-negative bacteria by the outer membrane composed of lipopolysaccharides [34]. Such a response was confirmed in this study as overall lime oil showed a higher effect against *S. aureus* 6538P than *E. coli* 8739.

#### 3.3.2. Halo Test

**Figure 5:**
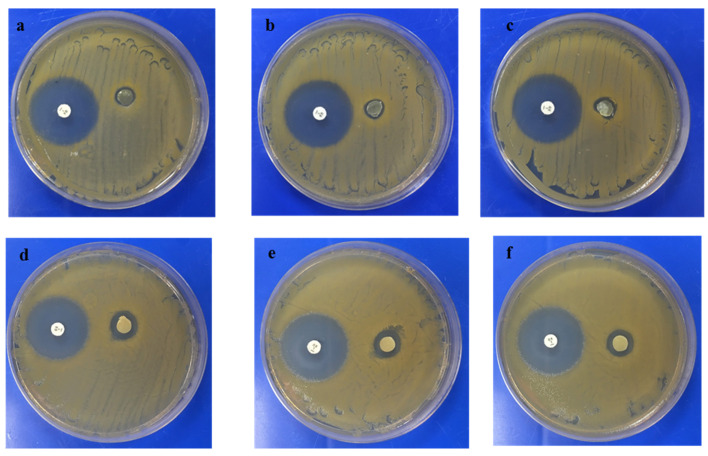
Halo test showing the antibacterial activity of aged lime oil containing P(3HO-*co*-3HD) films (**a**–**c**) and fresh lime oil containing films (**d**–**f**) against *S. aureus* 6538P (control: antibiotic disc containing 1 µg of oxacillin, left hand side).

The antimicrobial materials were tested against both *S. aureus* 6538P and *E. coli* 8739. Freshly prepared and aged films were analysed to investigate whether the material was able to preserve their antimicrobial efficacy. As it was discussed earlier, the content of lime oil decreased from 74 mg/g of copolymer for fresh film to 30 mg/g for the aged sample. The freshly prepared samples showed activity against *S. aureus* 6538P, inducing the formation of inhibition zones as shown in Figure 5 and Table 3. Moreover, the samples obtained after a one-year storage period still possessed activity against the microorganism, even though a 15% reduction of the zone of inhibition could be detected. By contrast, both the freshly prepared and aged films did not show activity against *E. coli* 8739, as reported in Table 3.

Limited research has been conducted on the incorporation of essential oils into PHAs and the studies conducted have focused only on SCL-PHAs. Films for antibacterial food packaging were produced using P(3HB-*co*-3HV) and two different essential oils such as oregano and clove, showing antibacterial activity against common food pathogens *Listeria innocua* 910 and *E. coli* 101 [35]. In another study, P(3HB-*co*-3HV) films were loaded with coconut fibres impregnated with oregano essential oil to produce composite antibacterial packaging materials, showing activity against *S. aureus* 6538P [36]. Alternatively, electrospinning has been investigated to produce fibrous mats based on P(3HB) or P(3HB-*co*-3HV) fibres containing essential oil. The natural compounds were either adsorbed onto the fibres after the spinning process (cinnamon, clove, oregano and oak bark) [37] or incorporated into the solution prior to electrospinning (rosemary and green tea extracts) [38]. The material produced showed activity against *Micrococcus luteus* 1569, *Serratia marcescens* 8587, *S. aureus* 6538P and *E. coli* 25922. Finally, Mendelez-Rodriguez *et al*. produced P(3HB-*co*-3HV) antibacterial fibres by loading eugenol essential oil onto mesoporous silica nanoparticles, followed by their incorporation into the fibres by electrospinning. The encapsulation was employed to maintain and protect the antibacterial effect of the agent, as essential oils are volatile compounds. The films obtained showed antibacterial activity against the *S. aureus* 6538P and *E. coli* 25922 up to 15 days after the day of fabrication and storage in a tightly closed system [39].

## 4. Conclusions

This study demonstrated the valorisation of sugarcane molasses, an agri-food by-product, through the production of a high-value MCL-PHA. The use of sugarcane molasses as the single carbon source and *Pseudomonas mendocina* CH50, a known MCL PHA producer, resulted in the synthesis of P(3HO-*co*-3HD) copolymer. P(3HO-*co*-3HD) is a polyester with low glass-transition and melting temperature, high elasticity and flexibility. In order to expand its potential applications, in this study, we explored the possibility of making P(3HO-*co*-3HD) antimicrobial through the incorporation of lime oil, an essential oil known for its antimicrobial activity. The antibacterial properties of the lime oil were investigated using the ISO 20776 against two bacterial strains, Gram-positive *S. aureus* 6538P and Gram-negative *E. coli* 8739. In line with general observations on the activity of essential oils, the lime oil used in this study was more active against *S. aureus* 6538P than *E. coli* 8739. Incorporation of the lime oil into the P(3HO-*co*-3HD) film had a minor effect on the material properties, slightly decreasing its crystallinity. As expected, the content of lime oil decreased with the storage due to migration out and evaporation from the polymer films. After one-year storage, the content of lime oil in the films decreased from *ca* 7 wt.% to 2.9 wt.%. Although the antimicrobial activity decreased after prolonged storage, both fresh and aged films of P(3HO-*co*-3HD) with incorporated lime oil showed activity against *S. aureus* 6538P. On the contrary, no antimicrobial activity was observed against *E. coli* 8739 for both fresh and aged films. The feasibility of imparting microbial activity to PHAs can broaden the application of this biodegradable polymer in several sectors, including coatings, adhesives, biodegradable rubbers, implantable materials. In the last sector, the developed P(3HO-*co*-3HD)/lime oil films could be employed as an antibacterial material for the regeneration of soft tissues, like skin.

## Figures and Tables

**Table 1:** Thermal properties of P(3HO-co-3HD) neat films and films with incorporated lime oil.

Sample	Thermal Properties		
	T_g_, °C	T_m_, °C ^a^	∆H, J/g
Neat film	−45.6	55.6	18.2
Fresh film with lime oil	−44.3	51.4	14.7
Aged film with lime oil	−40.8	50.5	15.8

^a^ Determined as the end of melting peak.

**Table 2:** Minimum inhibition concentration (MIC) and minimum bactericidal concentration (MBC) values of lime against *S. aureus* 6538P and *E. coli* 8739.

*S. aureus* 6538P		*E coli* 8739	
MIC (µL/mL)	MBC (µL/mL)	MIC (µL/mL)	MBC (µL/mL)
60	100	80	-

**Table 3:** Halo diameters showing the effect of P(3HO-*co*-3HD) films containing lime oil against *S. aureus* 6538P and *E. coli* 8739.

Sample	Zone of Inhibition (ZOI) (cm)	
	*S. aureus* 6538P	*E. coli* 8739
Aged P(3HO-*co*-3HD)/lime films	1.06 ± 0.02	-
Fresh P(3HO-*co*-3HD)/lime films	1.23 ± 0.05	0.13 ± 0.04
Control	3.13 ± 0.10 (oxacillin)	1.99 ± 0.09 (streptomycin)
